# Epiregulin (EREG) and Myocardin Related Transcription Factor A (MRTF-A) Form a Feedforward Loop to Drive Hepatic Stellate Cell Activation

**DOI:** 10.3389/fcell.2020.591246

**Published:** 2021-01-15

**Authors:** Xiaoyan Wu, Wenhui Dong, Tianyi Zhang, Haozhen Ren, Jinglin Wang, Longcheng Shang, Zhengyi Zhu, Wei Zhu, Xiaolei Shi, Yong Xu

**Affiliations:** ^1^Department of Hepatobiliary Surgery, The Affiliated Nanjing Drum Tower Hospital of Nanjing University Medical School, Nanjing, China; ^2^Hepatobiliary Institute, Nanjing University, Nanjing, China; ^3^Department of Anesthesiology, The Affiliated Drum Tower Hospital of Nanjing University Medical School, Nanjing, China; ^4^Key Laboratory of Targeted Intervention of Cardiovascular Disease, Collaborative Innovation Center for Cardiovascular Translational Medicine, and Center for Experimental Medicine, Department of Pathophysiology, Nanjing Medical University, Nanjing, China; ^5^Institute of Biomedical Research, Liaocheng University, Liaocheng, China

**Keywords:** transcription regulation, hepatic stellate cell, liver fibrosis, epiregulin, MRTF-A, SRF

## Abstract

Trans-differentiation of quiescent hepatic stellate cells (HSC) into myofibroblast cells is considered the linchpin of liver fibrosis. A myriad of signaling pathways contribute to HSC activation and consequently liver fibrosis. Epidermal growth factor (EGF) family of cytokines signal through the cognate receptor EGFR to promote HSC activation. In the present study we investigated the transcription regulation of epiregulin (EREG), an EGFR ligand, during HSC activation. We report that EREG expression was significantly up-regulated in activated HSCs compared to quiescent HSCs isolated from mice. In addition, there was an elevation of EREG expression in HSCs undergoing activation *in vitro*. Of interest, deficiency of myocardin-related transcription factor A (MRTF-A), a well-documented regulator of HSC trans-differentiation, attenuated up-regulation of EREG expression both *in vivo* and *in vitro*. Further analysis revealed that MRTF-A interacted with serum response factor (SRF) to bind directly to the EREG promoter and activate EREG transcription. EREG treatment promoted HSC activation *in vitro*, which was blocked by MRTF-A depletion or inhibition. Mechanistically, EREG stimulated nuclear trans-location of MRTF-A in HSCs. Together, our data portray an EREG-MRTF-A feedforward loop that contributes to HSC activation and suggest that targeting the EREG-MRTF-A axis may yield therapeutic solutions against liver fibrosis.

## Introduction

Liver fibrosis is a key pathophysiological process taking place in response to various acute and chronic hepatic injuries (Lee et al., 2015). Whereas spatiotemporally controlled liver fibrosis is instrumental to the amelioration of liver injury and restoration of liver function, excessive and/or prolonged liver fibrosis leads to architectural and functional damages to the liver and precipitates the development of such end-stage liver diseases as cirrhosis and hepatocellular carcinoma (Barry et al., 2020). Liver fibrosis can occur following the challenge of a myriad of injurious stimuli including pathogens, toxins, corrosive chemicals, and metabolites. Regardless of the specific triggering factor, myofibroblasts are considered the major effector cell type for liver fibrosis (Kisseleva, 2017). Myofibroblasts possess the characteristics of both muscle cells and fibroblast cells being able to contract and cover the wound and produce and lay down extracellular matrix proteins. The origins from which myofibroblasts arise during liver fibrosis have been a subject matter receiving extensive investigations. Recently lineage fate-mapping experiments have been determined that an overwhelming majority (>90%) of myofibroblasts in the liver are derived from hepatic stellate cells (HSC) that express lecithin retinol acyltransferase (Lrat), a supposedly HSC lineage-specific marker gene (Mederacke et al., 2013). Under physiological settings, quiescent HSCs primarily function as a deposit site for lipids and vitamin A; upon exposure to a pro-fibrogenic microenvironment, HSCs undergo trans-differentiation and become myofibroblasts. *In vitro* cultured HSCs can also be educated to switch to a myofibroblast-like phenotype by a host of growth factors including transforming growth factor (TGF-β) and platelet-derived growth factor (PDGF) (Hou and Syn, 2018).

Signaling through epidermal growth factor receptor (EGFR) has been shown to contribute to HSC activation and liver fibrosis. Scheving et al. have demonstrated that genetic deletion of EGFR attenuates CCl_4_ induced liver fibrosis in mice (Scheving et al., 2016). Consistently, pharmaceutical inhibition of EGFR is associated with amelioration of liver fibrosis in different murine models (Fuchs et al., 2014; Liang et al., 2018). Previously, Perugorria et al. have shown the amphiregulin (AR), a ligand for EGFR, plays critical roles in HSC activation and liver fibrosis: AR treatment robustly promotes HSC activation *in vitro* whereas AR deletion protects the mice from CCl_4_-induced liver fibrosis (Perugorria et al., 2008). Epiregulin, encoded by *EREG*, is an EGFR ligand that shares significant homology with amphiregulin (Riese and Cullum, 2014). Whether EREG can contribute to HSC activation remains undetermined.

Mounting evidence suggests that myocardin-related transcription factor A (MRTF-A) plays a pivotal role promoting the differentiation of myofibroblasts in multiple organs (Small, 2012). MRTF-A was initially characterized as a co-factor for serum response factor (SRF) to activate the transcription of muscle-lineage specific genes (Wang et al., 2002). MRTF-A can shuttle between the cytoplasm and the nucleus depending on cytoskeletal reshuffling (Olson and Nordheim, 2010). Previously we have reported that MRTF-A regulates liver fibrosis by transcriptionally programming HSC activation (Fan et al., 2015; Tian et al., 2015, 2016). Here we report that EREG expression is up-regulated during HSC activation both *in vivo* and *in vitro*. MRTF-A interacts with SRF to directly bind to the EREG promoter and activate EREG transcription. Reciprocally, EREG contributes to HSC activation by promoting nuclear trans-location of MRTF-A. Therefore, targeting the EREG-MRTF-A axis may yield therapeutic solutions against liver fibrosis.

## Materials and Methods

### Animals

All animal protocols were reviewed and approved the intramural Ethics Committee on Humane Treatment of Laboratory Animals of Nanjing Medical University. MRTF-A knockout (KO) mice were originally obtained from Steve Morris at St Jude Hospital (Sun et al., 2006). To induce liver fibrosis, MRTF-A KO mice and wild type (WT) littermates were injected with CCl_4_ (1.0 mL/kg as 50% vol/vol), or injected with thioacetamide (TAA, 100 mg/kg), or subjected to bile duct ligation (BDL) as previously described (Li et al., 2019a, c; Lu et al., 2019).

### Cell Culture, Plasmids, and Transient Transfection

Immortalized human HSC (LX-2) were maintained in DMEM supplemented with 10% FBS as previously described (Kong et al., 2019a, b). Primary HSC were isolated and maintained as previously described (Li et al., 2019b). Briefly, the animals were anesthetized by intraperitoneal injection with ketamine-xylazine. A laparotomy was performed and the portal vein was cut to allow retrograde perfusion with pronase (Sigma Aldrich, St. Louis, MO, United States) and collagenase (Roche, Germany) containing solutions. HSCs were isolated from the non-parenchymal fraction by 9.7% Nycodenz gradient centrifugation. Isolated HSCs were seeded in plastic culture dishes and allowed to undergo spontaneous activation. RNA targeting SRF (GAUGGAGUUCAUCGACAACAA) was purchased from Dharmacon. Recombinant TGF-β (100-21) was purchased from Peprotech. Recombinant EREG (1195-EP-025) was purchased from R&D. CCG-1423 (S7719) was purchased from Selleck. Full-length EREG promoter-luciferase construct (−1345/+118) and MRTF-A expression construct have been previously described (Kyotani et al., 2018; Mao et al., 2020b). Truncated and mutated EREG promoter-luciferase constructs were prepared with the QuikChange mutagenesis kit (Agilent). Conditioned media were harvested as previously described (Li et al., 2020a, b). Briefly, the cells were switched to and incubated with serum-free media overnight. The next day, the media were collected, centrifuged at 4,000 × *g* for 30 min at 4°C using 3-kDa MW cut-off filter units (Millipore) and sterilized through a 0.4-μm filter. Transient transfections were performed with Lipofectamine 2000. Luciferase activities were assayed 24–48 h after transfection using a luciferase reporter assay system (Promega) as previously described (Yang et al., 2019a, b).

### Enzyme-Linked Immunosorbent Assay (ELISA)

Secreted epiregulin levels were measured using a commercially available ELISA (LS-F5753, Lifespan Biosciences) per vendor’s recommendations.

### Protein Extraction and Western Blot

Whole cell lysates were obtained by re-suspending cell pellets in RIPA buffer (50 mM Tris pH7.4, 150 mMNaCl, 1% Triton X-100) with freshly added protease inhibitor (Roche) as previously described (Fan et al., 2020). Nuclear proteins were extracted using the NE-PER Kit (Pierce) following manufacturer’s recommendation (Mao et al., 2020a). Western blot analyses were performed with anti-MRTF-A (Santa Cruz, sc-32909), anti-SRF (Cell Signaling Technology, 5147), anti-α-tubulin (Sigma, T6074), anti-Lamin A/C (Proteintech, 10298-1), anti-α-SMA (Abcam, ab5694), and anti-β-actin (Sigma, A1978). For densitometrical quantification, densities of target proteins were normalized to those of β-actin as previously described (Lv et al., 2020; Wu et al., 2020). Data are expressed as relative protein levels compared to the control group which is arbitrarily set as 1.

### RNA Isolation and Real-Time PCR

RNA was extracted with the RNeasy RNA isolation kit (Qiagen). Reverse transcriptase reactions were performed using a SuperScript First-strand Synthesis System (Invitrogen) as previously described (Zhao et al., 2019; Dong et al., 2020). Real-time PCR reactions were performed on an ABI Prism 7,500 system with the following primers: human EREG, 5′-ACGTGTGGCTCAAGTGTCAA-3′ and 5′-CACTTCACACC TGCAGTAGTTT-3′; mouse Ereg, 5′-TGCTTTGTCTAGGTT CCCACC-3′ and 5′-GGCGGTACAGTTATCCTCGG-3′; human COL1A2, 5′-GTGGCAGTGATGGAAGTGTG-3′ and 5′-AGGA CCAGCGTTACCAACAG-3′; human ACTA2, 5′-CTATGCC TCTGGACGCACAACT-3′ and 5′-CAGATCCAGACGCAT GATGGCA-3′.Ct values of target genes were normalized to the Ct values of housekeekping control gene (18s, 5′-CGCGGTTCTATTTTGTTGGT-3′ and 5′-TCGTCTTCG AAACTCCGACT-3′ for both human and mouse genes) using the ΔΔCt method and expressed as relative mRNA expression levels compared to the control group which is arbitrarily set as 1.

### Chromatin Immunoprecipitation

Chromatin Immunoprecipitation (ChIP) assays were performed essentially as described before (Sun et al., 2020). In brief, chromatin in control and treated cells were cross-linked with 1% formaldehyde. Cells were incubated in lysis buffer (150 mMNaCl, 25 mM Tris pH 7.5, 1% Triton X-100, 0.1% SDS, 0.5% deoxycholate) supplemented with protease inhibitor tablet and PMSF. DNA was fragmented into ∼200 bp pieces using a Branson 250 sonicator. Aliquots of lysates containing 200 μg of protein were used for each immunoprecipitation reaction with anti-MRTF-A (Santa Cruz, sc-32909), anti-SRF (Cell Signaling Technology, 5147), or pre-immune IgG. For re-ChIP, immune complexes were eluted with the elution buffer (1% SDS, 100 mM NaCO_3_), diluted with the re-ChIP buffer (1% Triton X-100, 2 mM EDTA, 150 mMNaCl, 20 mM Tris pH 8.1), and subjected to immunoprecipitation with a second antibody of interest.

### Immunofluorescence Microscopy

Immunofluorescence staining was performed as previously described. The cells were fixed with 4% formaldehyde, permeabilized with TBST (0.25% Triton X-100, 150 mM NaCl, 50 mM Tris pH7.4), blocked with 5% BSA, and incubated with indicated primary antibodies overnight. After several washes with PBS, cells were incubated with FITC-labeled secondary antibodies (Jackson) for 30 min. DAPI (Sigma) was added and incubated with cells for 5 min prior to observation. Immunofluorescence was visualized on a co-focal microscope (LSM 710, Zeiss). For each group, at least 10 fields were counted.

### Statistical Analysis

One-way ANOVA with *post hoc* Scheff’e analyses were performed by SPSS software (IBM SPSS v18.0, Chicago, IL, United States). Unless otherwise specified, values of *p* < 0.05 were considered statistically significant.

## Results

### EREG Expression Is Up-Regulated in Activated HSCs

**FIGURE 1:**
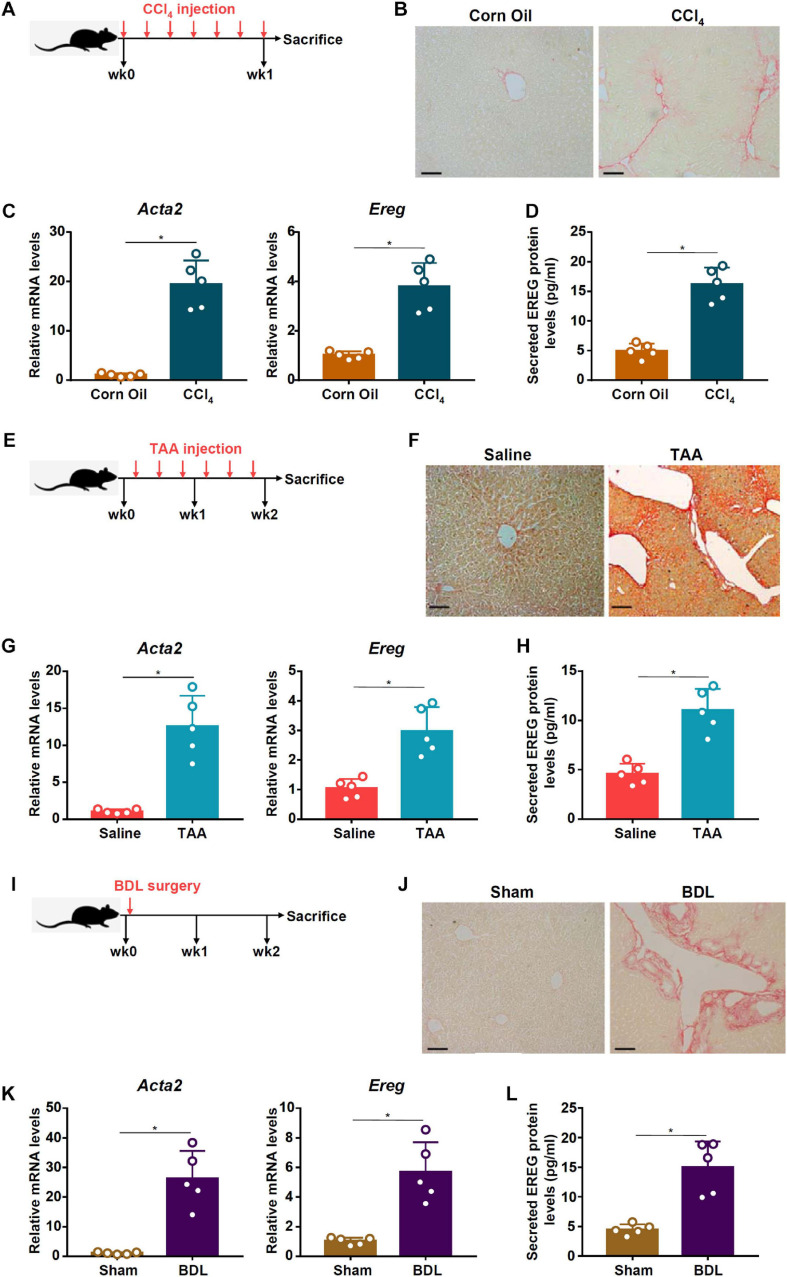
EREG expression is up-regulated in activated HSCs in vivo. **(A–D)** C57/BL6 mice were injected with CCl_4_ or corn oil for 7 days. Scheme of protocol **(A)**. Representative images of picrosirius red staining **(B)**. Primary HSCs were isolated from the mice and EREG expression levels were examined by qPCR **(C)** and ELISA **(D)**. *N* = 5 mice for each group. **(E–H)** C57/BL6 mice were injected with TAA or saline for 2 weeks. Scheme of protocol **(E)**. Representative images of picrosirius red staining **(F)**. Primary HSCs were isolated from the mice and EREG expression levels were examined by qPCR **(G)** and ELISA **(H)**. *N* = 5 mice for each group. **(I–L)** C57/BL6 mice were subjected to the BDL procedure or the sham surgery. The mice were sacrificed 2 weeks after the surgery and primary HSCs were isolated. Scheme of protocol **(I)**. Representative images of picrosirius red staining **(J)**. EREG expression levels were examined by qPCR **(K)** and ELISA **(L)**. *N* = 5 mice for each group.

Previously it has been shown that amphiregulin (AR), an EGFR ligand closely related to epiregulin (EREG), is activated during HSC trans-differentiation and contributes to liver fibrosis (Perugorria et al., 2008). We asked whether EREG expression levels might be altered during HSC activation. To this end, C57/BL6 mice were injected with CCl_4_ to induce liver fibrosis (Figure 1A). Picrosirius red staining showed significant liver fibrosis in the CCl_4_-injected mice compared to the vehicle-injected mice (Figure 1B). Primary HSCs were isolated from the mice with liver fibrosis and from the control mice receiving injection with corn oil. As shown in Figure 1C, expression of α-SMA (*Acta2*), a myofibroblast marker, was significantly up-regulated, as measured by qPCR, in the activated HSCs compared to the quiescent HSCs; a similar up-regulation of *Ereg* expression was detected in the HSCs isolated from the fibrotic livers compared to those isolated from the control livers. ELISA measurements confirmed that EREG protein levels were also up-regulated in the activated HSCs compared to the quiescent HSCs (Figure 1D). Next, liver fibrosis was induced in mice by injection with thioacetamide (TAA, Figure 1E). Picrosirius red staining showed significant liver fibrosis in the TAA-injected mice compared to the vehicle-injected mice (Figure 1F). Again, primary HSCs isolated from the fibrotic livers displayed higher levels of *Acta2* and *Ereg* than those isolated from the control livers (Figure 1G). A similar increase in EREG protein levels was detected by ELISA (Figure 1H). Finally, in a third model of liver fibrosis in which the mice were subjected to the BDL surgery (Figure 1I), picrosirius red staining showed significant liver fibrosis in the BDL mice compared to the sham-operated mice (Figure 1J). qPCR (Figure 1K) and ELISA (Figure 1L) assays showed that EREG expression levels were up-regulated during HSC activation *in vivo*.

**FIGURE 2:**
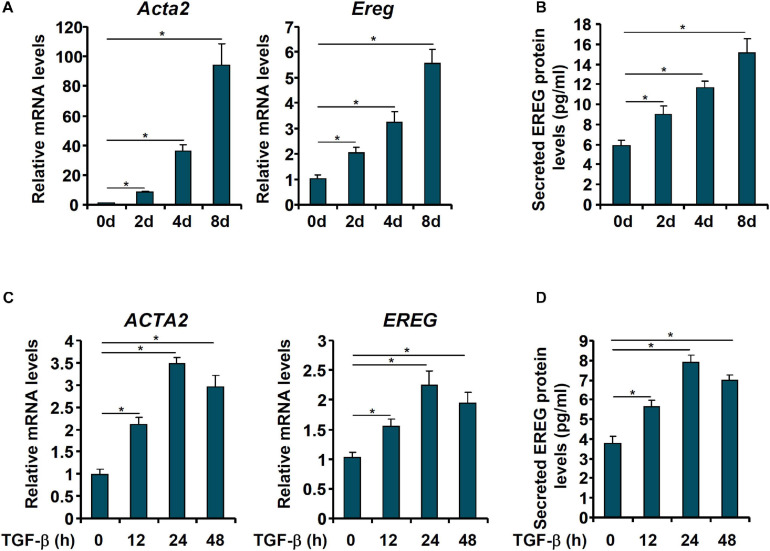
EREG expression is up-regulated in activated HSCs in vitro. **(A,B)** Primary HSCs were isolated from C57/BL6 mice and underwent spontaneous activation by *in vitro* culture. The cells were harvested at indicated time points and EREG expression levels were examined by qPCR and ELISA. **(C,D)** LX-2 cells were treated with or without TGF-β (5 ng/ml) and harvested at indicated time points. EREG expression levels were examined by qPCR and ELISA.

We then evaluated the changes in epiregulin expression in cell models of liver fibrosis. In the first model, primary HSCs were isolated from C57/BL mice and allowed to undergo spontaneous activation *in vitro*. When the cells were harvested at different time points following their isolation, it was observed that *Ereg* expression was progressively up-regulated mirroring the changes in *Acta2* expression (Figures 2A,B). In the second model, LX-2 cells were treated with TGF-β, a well-documented pro-fibrogenic growth factor. Epiregulin expression was significantly up-regulated by TGF-β treatment mirroring the increase in α-SMA expression (Figures 2C,D). Taken together, these data suggest a positive correlation between epiregulin and hepatic stellate cell activation *in vivo* and *in vitro*.

### EREG Stimulates Pro-fibrogenic Gene Expression in Hepatic Stellate Cells

**FIGURE 3:**
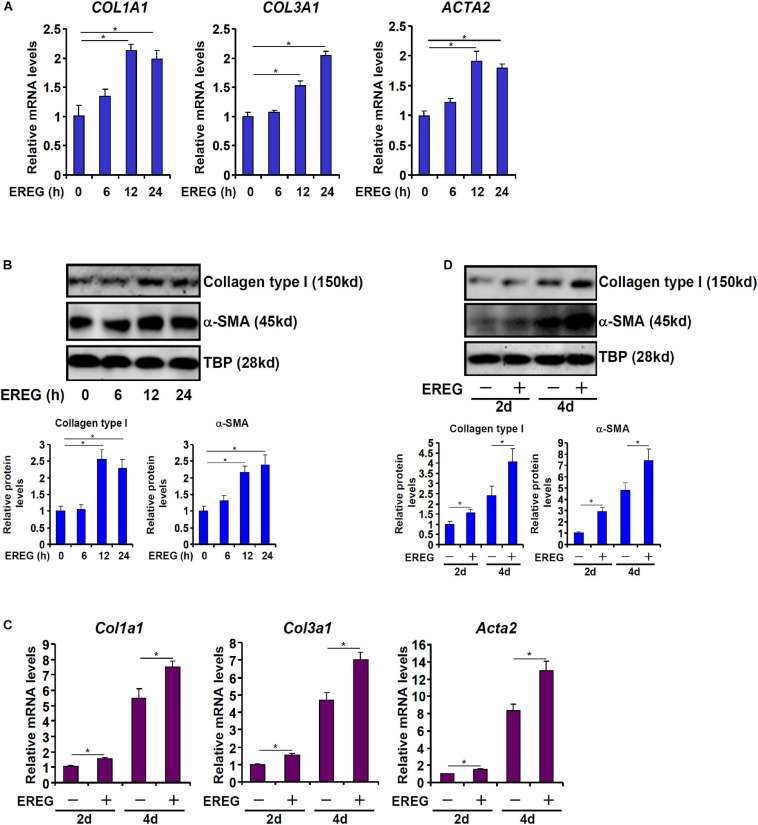
EREG treatment stimulates the expression of fibrogenic genes in HSCs. **(A,B)** LX-2 cells were treated with or without EREG (5 ng/ml) and harvested at indicated time points. Gene expression levels were examined by qPCR and Western blotting. Quantification was performed with Image Pro based on three independent experiments. **(C,D)** Primary HSCs were isolated from C57/BL6 mice and underwent spontaneous activation by *in vitro* culture in the presence or absence of EREG. Gene expression levels were examined by qPCR and Western blotting. Quantification was performed with Image Pro based on three independent experiments.

Next, we evaluated the effect of epiregulin on pro-fibrogenic gene expression in hepatic stellatecells. Treatment with epiregulin led to a small but appreciable increase in pro-fibrogenic gene expression in LX-2 cells as early as 6 h after the treatment. Induction of pro-fibrogenic genes by epiregulin treatment were detected at 12 and 24 h by qPCR (Figure 3A) and Western blotting (Figure 3B). We also examined the effect of epiregulin treatment on pro-fibrogenic gene expression in spontaneously activated primary HSCs. As shown in Figures 3C,D, the addition of epiregulin augmented the up-regulation of pro-fibrogenic genes as primary HSCs transition from a quiescent state to an activated state. To make a broad point that HSCs can produce and release factors to promote/sustain activation in a feedforward fashion, conditioned media (CM) were harvested from spontaneously activated HSCs to treat quiescent HSCs. Indeed, quiescent HSCs treated with the CM transitioned into an activated state faster than the HSCs cultured in regular media (Supplementary Figure 1). Therefore, it appears that epiregulin may promote the activation of HSCs via an autocrine pathway *in vitro*.

### MRTF-A Deficiency Results in Down-Regulation of EREG Expression in Hepatic Stellate Cells

**FIGURE 4:**
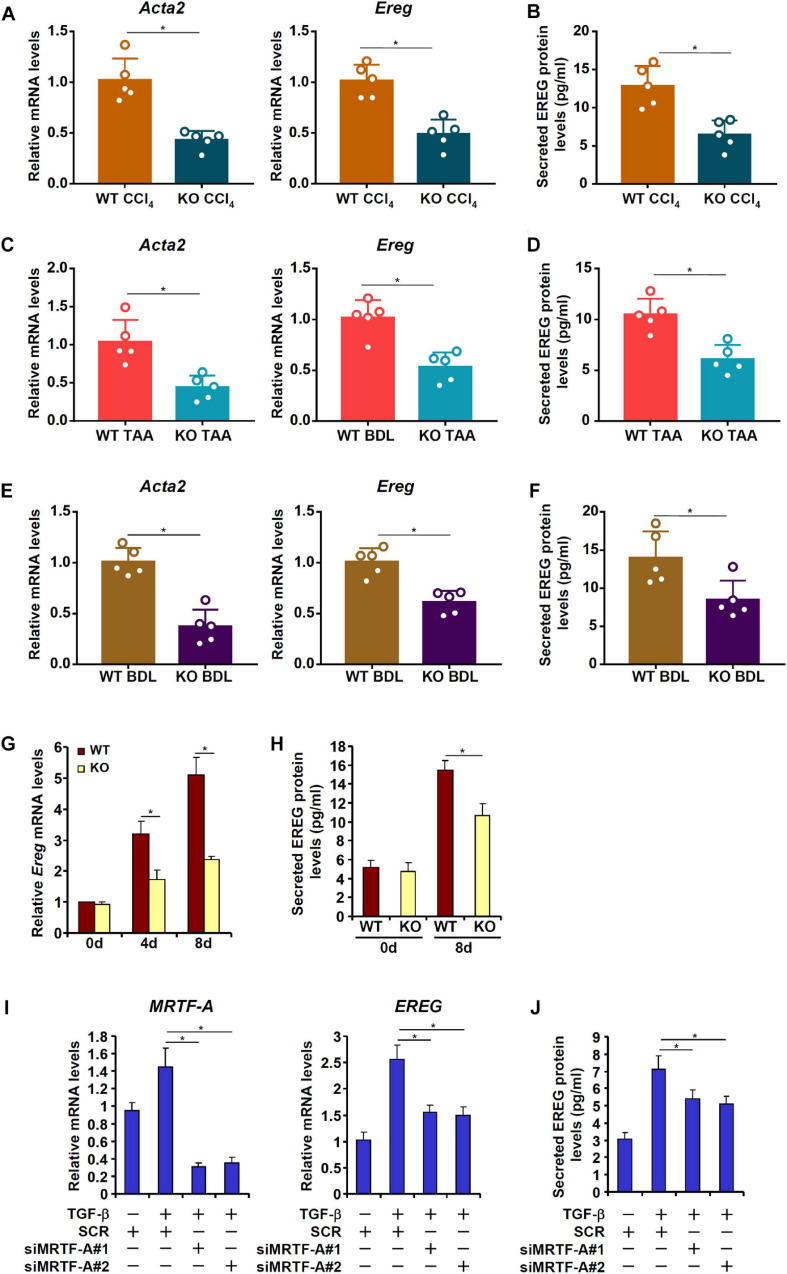
MRTF-A deficiency attenuates EREG activation in HSCs. **(A,B)** WT and MRTF-A KO mice were injected with CCl_4_ for 7 days. Primary HSCs were isolated from the mice and EREG expression levels were examined by qPCR and ELISA. *N* = 5 mice for each group. **(C,D)** WT and MRTF-A KO mice were injected with TAA for 2 weeks. Primary HSCs were isolated from the mice and EREG expression levels were examined by qPCR and ELISA. *N* = 5 mice for each group. **(E,F)** WT and MRTF-A KO mice were subjected to the BDL procedure. The mice were sacrificed 2 weeks after the surgery and primary HSCs were isolated. EREG expression levels were examined by qPCR and ELISA. *N* = 5 mice for each group. **(G,H)** Primary HSCs were isolated from WT and MRTF-A KO mice and underwent spontaneous activation for 7 days. EREG expression levels were examined by qPCR and ELISA. **(I,J)** LX-2 cells were transfected with siRNAs targeting MRTF-A or scrambled siRNAs (SCR) followed by treatment with TGF-β (5 ng/ml) for 24 h. EREG expression levels were examined by qPCR and ELISA.

MRTF-A is key determinant of myofibroblast maturation (Small, 2012). We have previously shown that MRTF-A deletion in mice attenuated liver fibrosis induced by TAA injection (Tian et al., 2016), by CCl_4_ injection (Tian et al., 2015), or by the BDL procedure (Fan et al., 2015). Consistently, primary HSCs isolated from the MRTF-A KO mice exhibited reduced expression of *Acta2* compared to the WT mice following TAA injection (Figure 4A), CCl_4_ injection (Figure 4C) or the BDL procedure (Figure 4E). Of interest, MRTF-A deficiency comparably decreased epiregulin expression in all three models as measured by qPCR (Figures 4A,C,E) and ELISA (Figures 4B,D,F). In keeping with these observations, induction of epiregulin expression was much more tepid during spontaneous activation of primary HSCs isolated from MRTF-A KO mice than from WT mice (Figures 4G,H). Finally, knockdown of MRTF-A by siRNAs repressed induction of epiregulin expression by TGF-β treatment in LX-2 cells (Figures 4I,J). In contrast, knockdown of MRTF-B, a closely related MRTF-A sibling, did not alter epiregulin expression in either LX-2 cells or primary HSCs (Supplementary Figure 2).

### MRTF-A Activates EREG Transcription by Interacting With SRF

**FIGURE 5:**
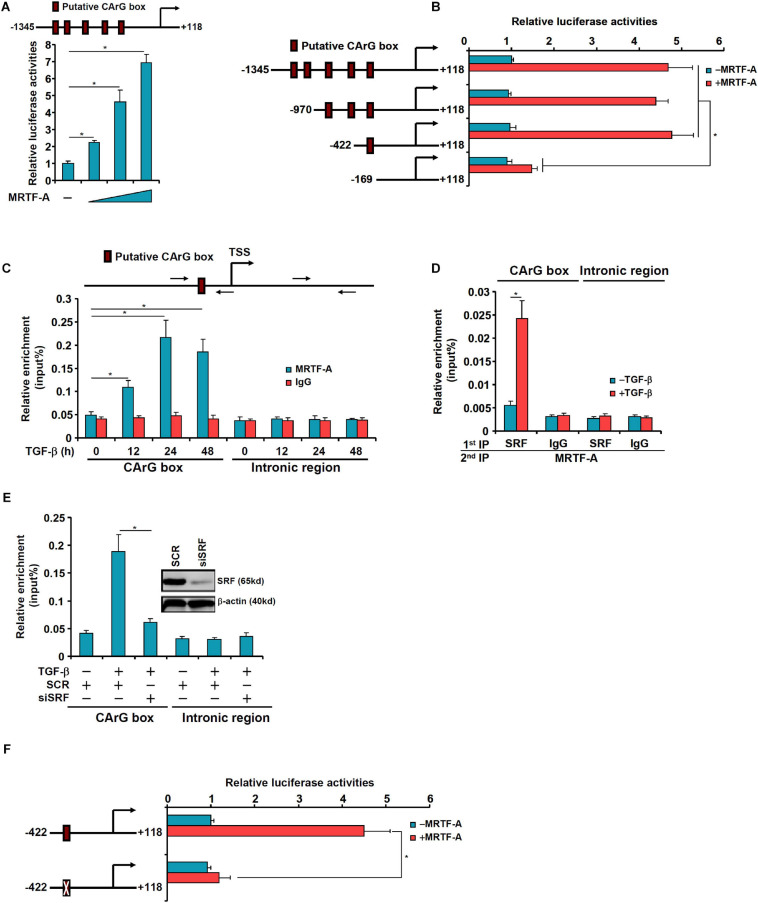
MRTF-A directly activates EREG transcription. **(A)** A human EREG promoter-luciferase constructs (–1345/ + 118) were transfected into LX-2 cells with or without MRTF-A. Luciferase activities were normalized by protein concentration and GFP fluorescence. **(B)** Wild type or truncated EREG promoter-luciferase constructs were transfected transfected into LX-2 cells with or without MRTF-A. Luciferase activities were normalized by protein concentration and GFP fluorescence. **(C)** LX-2 cells were treated with or without TGF-β (5 ng/ml) and harvested at indicated time points. ChIP assays were performed with anti-MRTF-A or IgG. **(D)** LX-2 cells were treated with or without TGF-β (5 ng/ml) for 24 h. Re-ChIP assay was performed with indicated antibodies. **(E)** LX-2 cells were transfected with siRNA targeting SRF or scrambled siRNAs (SCR) followed by treatment with TGF-β (5 ng/ml) for 24 h. ChIP assays were performed with anti-MRTF-A. **(F)** Wild type or CArG mutated EREG promoter-luciferase construct were transfected into LX-2 cells with or without MRTF-A. Luciferase activities were normalized by protein concentration and GFP fluorescence.

We asked whether MRTF-A might regulate epiregulin expression at the transcriptional level. To test this hypothesis, an *EREG* promoter-luciferase fusion construct (−1345/ + 118) was transfected into LX-2 cells. Over-expression of MRTF-A dose-dependently up-regulated the *EREG* promoter activity (Figure 5A). A string of CArG box elements were identified within the *EREG* promoter (Figure 5A). Inward deletions introduced to the *EREG* promoter progressively removed the CArG box elements; the removal of the four more distal CArG boxes retained the responsiveness of the *EREG* promoter to MRTF-A over-expression whereas the removal of the most proximal CArG box rendered the *EREG* promoter inactive (Figure 5B). Several lines of additional evidence suggest that MRTF-A relies on the proximal CArG box to activate *EREG* transcription. ChIP assay showed that TGF-β treatment enhanced the association of MRTF-A with the proximal *EREG* promoter surrounding the innermost CArG box, but not with the intronic region, in LX-2 cells (Figure 5C). Similarly, association of MRTF-A with the *Ereg* promoter was stronger in activated primary HSCs compared to quiescent primary HSCs (Supplementary Figure 3). Re-ChIP assay confirmed that TGF-β treatment promoted the formation of an SRF-MRTF-A complex on the proximal *EREG* promoter (Figure 5D). Depletion of SRF with siRNA completely disrupted the binding of MRTF-A to the *EREG* promoter (Figure 5E). Finally, mutation of the most proximal CArG box abrogated the induction of the *EREG* promoter by MRTF-A (Figure 5F).

### EREG Regulates HSC Activation by Promoting Nuclear Trans-Location of MRTF-A

**FIGURE 6:**
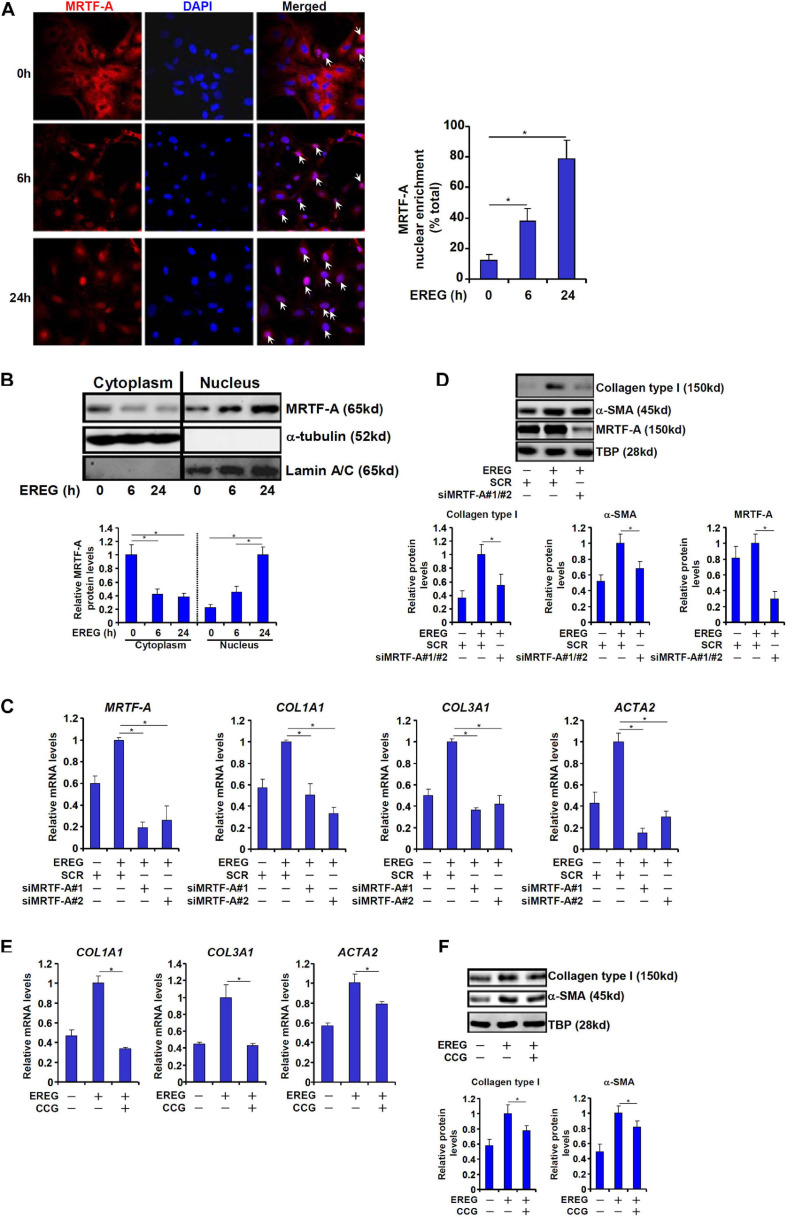
EREG regulates HSC activation through MRTF-A. **(A)** LX-2 cells were treated with EREG (10 ng/ml) and harvested at indicated time points. MRTF-A localization was examined by immunofluorescence staining. **(B)** LX-2 cells were treated with EREG (10 ng/ml) and harvested at indicated time points. MRTF-A localization was examined by cell fractionation followed by Western blotting. Quantification was performed with Image Pro based on three independent experiments. **(C,D)** LX-2 cells were transfected with siRNAs targeting MRTF-A or scrambled siRNA (SCR) treated with recombinant EREG (10 ng/ml) for 24 h. Gene expression was examined by qPCR and Western. Quantification was performed with Image Pro based on three independent experiments. **(E,F)** LX-2 cells were treated with recombinant EREG (10 ng/ml) in the presence or absence of CCG for 24 h. Gene expression was examined by qPCR and Western. Quantification was performed with Image Pro based on three independent experiments.

Finally, we asked whether epiregulin could reciprocally influence MRTF-A activity. MRTF-A typically shuttles between the cytoplasm and the nucleus (Olson and Nordheim, 2010). Immunofluorescence staining showed that a large fraction of MRTF-A resides in the cytoplasm with only ∼10% located to the nucleus in LX-2 cells under normal conditions. When exposed to epiregulin treatment, MRTF-A started migrating into the nucleus: at 6 h following epiregulin treatment ∼35% of all MRTF-A proteins whereas at 24 h over 75% of all MRTF-A proteins were detected in the nucleus (Figure 6A). Similar discoveries were made by cell fractionation/Western blotting (Figure 6B). Conversely, induction of pro-fibrogenic genes by epiregulin treatment in LX-2 cells was markedly suppressed by MRTF-A knockdown at both mRNA (Figure 6C) and protein (Figure 6D) levels. Finally, inhibition of MRTF-A activity by CCG-1423 also dampened the up-regulation of pro-fibrogenic genes by epiregulin treatment (Figures 6E,F).

## Discussion

Activation of HSCs, a hallmark event in liver fibrosis, is programmed by a complex web of signaling cascades that relay the pro-fibrogenic cues to the nucleus, where profound changes in gene expression contribute to the transition of quiescent HSCs to mature myofibroblasts. In the present report, we provide data to show that epiregulin (EREG), belonging to the EGF family of growth factors, is a key regulator of HSC activation. Our data indicate that exposure of cultured HSCs to recombinant epiregulin accelerated the production of pro-fibrogenic genes (Figure 3). This is consistent with previously reported observations that other members of the EGF family including EGF (Lin and Chen, 2008), heparin-bound EGF (HB-EGF) (Takemura et al., 2013), FGF (Wang et al., 2020), and amphiregulin (Perugorria et al., 2008) all contribute to HSC trans-differentiation and liver fibrosis. Of note, all four EGF ligands signal through the same receptor (EGFR) (Riese and Cullum, 2014). Although existing evidence supports the argument that treatment with individual EGF growth factors is sufficient to promote HSC activation at least *in vitro*, the *in vivo* requirement/redundancy for each one of these proteins in the development of liver fibrosis is not clear. Deletion of either amphiregulin (AREG) or HB-EGF in mice leads to attenuation of liver fibrosis, suggesting that these growth factors may elicit different signaling cascades and downstream events to promote HSC maturation and fibrogenesis so that the loss of one EGF ligand cannot be fully compensated by other family members. Global EREG-null mice are viable and display no overt gross or liver abnormalities under physiological conditions (Lee et al., 2004). It remains to be ascertained whether these epiregulin deficient mice would phenocopy the AREG^–/–^ mice and the HB-EGF^–/–^ mice in models of liver fibrosis.

We show here that up-regulation of EREG expression during HSC activation *in vivo* and *in vitro* is mediated at the transcriptional level by MRTF-A, a well-established pro-fibrogenic molecule (Small, 2012). MRTF-A appears to activate EREG transcription by interacting with SRF and binding to one of the CArG boxes located on the proximal EREG promoter. Of interest, expression of other EGF family members has been shown to be regulated by SRF. For instance, differential regulation of neuregulin 1 (NRG1) in schizophrenia is controlled by several 5′ SNPs that create/abolish binding sites for a string of transcription factors including SRF (Law et al., 2006). On the other hand, SRF can be placed downstream of the EGFR signaling pathway. Augmented SRF activity by EGFR signaling is considered a paradigm in the pathogenesis of multiple cancers (Wee and Wang, 2017). More recently, Stern and colleagues have found that muscular conditional deletion of EGFR, which presumably blocks the signal transduction initiated by EGF, AREG, and EREG, protects the mice from diabetic complications, which is accompanied by changes in gene expression patterns reminiscent of suppressed SRF activity suggesting that EGFR likely regulates muscle cell behavior through stimulating SRF activity (Stern et al., 2020). It would be of great interest to delineate whether a reciprocal regulatory relationship exists between SRF and EREG in the process of HSC activation.

Our data suggest that EREG promotes HSC activation at least in part by inducing MRTF-A nuclear translocation (Figure 6). Sub-cellular localization of MRTF-A is known to be regulated by its post-translational modifications. One of the best characterized modifications of MRTF-A is serine/threonine phosphorylation. The Treisman laboratory has systemically profiled the dynamic alteration of MRTF-A phosphorylation status in response to serum withdrawal/re-addition in fibroblasts uncovering a total of 26 putative S/T residues subjected to phosphorylation (Panayiotou et al., 2016). It is noteworthy that three out of the 26 sites, including S98, T545, and S549, are regulated by EGF stimulation in Hela cells (Olsen et al., 2006). Whereas mutation of T545/S549 that renders the two sites un-phosphorylatable did not impact the cytoplasm-nucleus shuttling of MRTF-A, S98 phosphorylation is critically required for its nuclear accumulation upon serum stimulation (Panayiotou et al., 2016). Thus, it is possible that EREG might promote MRTF-A nuclear translocation by inducing S98 phosphorylation. This hypothesis certainly deserves further investigation.

In summary, we report an epiregulin-MRTF-A feedforward loop that contributes to HSC activation. At least two issues need to be addressed in future studies. First, whether this feedforward loop is relevant *in vivo* needs to be verified in animal models of liver fibrosis. Second, the mechanism by which epiregulin regulates MRTF-A activity needs to explored in depth. The current data, however, do provide sufficient rationale for designing small-molecule compounds that can blockade this EREG-MRTF-A loop in the intervention of liver fibrosis.

## Data Availability Statement

The original contributions presented in the study are included in the article/Supplementary Material, further inquiries can be directed to the corresponding author/s.

## Ethics Statement

The animal study was reviewed and approved by the Nanjing Medical University Ethics Committee on Humane Treatment of Experimental Animals.

## Author Contributions

XS and WZ conceived the project and secured funding and provided the supervision. XW, WD, and TZ designed the experiments. XW, WD, TZ, HR, JW, LS, and ZZ performed experiments and collected the data. YX wrote the manuscript. All authors contributed to the article and approved the submitted version.

## Conflict of Interest

The authors declare that the research was conducted in the absence of any commercial or financial relationships that could be construed as a potential conflict of interest.
