# Supplementary material for: Epiregulin (EREG) and Myocardin Related Transcription Factor A (MRTF-A) Form a Feedforward Loop to Drive Hepatic Stellate Cell Activation
Source: Front Cell Dev Biol. 2021 Jan 15;8:591246. doi: 10.3389/fcell.2020.591246 (PMC7843934; doi:10.3389/fcell.2020.591246)
Supplement: Supplementary file 1 [file Table_1.DOCX]

**Wu XY et al: *Epiregulin (EREG) and myocardin related transcription factor A (MRTF-A) form a feedforward loop to drive hepatic stellate cell activation***

**Online supplementary material**

**Fig.S1**: Conditioned media (CM) were harvested from primary murine HSCs having undergone spontaneous activation for 4d and 8d, respectively. Quiescent primary murine HSCs were treated with the 4d CM or 8d CM for 48h. Pro-fibrogenic genes were examined by qPCR.

**Figure S2**: (**A**) LX-2 cells were transfected with siRNAs targeting MRTF-B or scrambled siRNAs (SCR) followed by treatment with TGF-β (5ng/ml) for 24 hours. EREG expression levels were examined by qPCR. (**B**) Primary murine HSCs were transfected with siRNAs targeting MRTF-B or scrambled siRNAs (SCR) followed by spontaneous activation in vitro for 4d. EREG expression levels were examined by qPCR.

**Figure S3**: Primary HSCs were isolated from C57/B6 mice and allowed to undergo spontaneous activation in vitro. The cells are harvested at indicated time points and ChIP assay was performed with anti-MRTF-A or IgG.
